# Laboratory capacity building for the International Health Regulations (IHR[2005]) in resource-poor countries: the experience of the African Field Epidemiology Network (AFENET)

**DOI:** 10.1186/1471-2458-10-S1-S8

**Published:** 2010-12-03

**Authors:** Monica Musenero Masanza, Ndlovu Nqobile, David Mukanga, Sheba Nakacubo Gitta

**Affiliations:** 1 African Field Epidemiology Network, Plot 23 Mackenzie Vale. P.O. Box 12874, Kampala

## Abstract

Laboratory is one of the core capacities that countries must develop for the implementation of the International Health Regulations (IHR[2005]) since laboratory services play a major role in all the key processes of detection, assessment, response, notification, and monitoring of events. While developed countries easily adapt their well-organized routine laboratory services, resource-limited countries need considerable capacity building as many gaps still exist. In this paper, we discuss some of the efforts made by the African Field Epidemiology Network (AFENET) in supporting laboratory capacity development in the Africa region. The efforts range from promoting graduate level training programs to building advanced technical, managerial and leadership skills to in-service short course training for peripheral laboratory staff. A number of specific projects focus on external quality assurance, basic laboratory information systems, strengthening laboratory management towards accreditation, equipment calibration, harmonization of training materials, networking and provision of pre-packaged laboratory kits to support outbreak investigation. Available evidence indicates a positive effect of these efforts on laboratory capacity in the region. However, many opportunities exist, especially to support the roll-out of these projects as well as attending to some additional critical areas such as biosafety and biosecuity. We conclude that AFENET’s approach of strengthening national and sub-national systems provide a model that could be adopted in resource-limited settings such as sub-Saharan Africa.

## Introduction

In today’s interconnected world, the risk of international spread of infectious diseases has greatly increased. This was well illustrated by the rapid spread of the recent influenza (H1N1) and severe acute respiratory syndrome (SARS) epidemics, where all continents were quickly threatened by an emergent pathogen in one corner of the world. The purpose of International Health Regulations (IHR[2005]) is “to prevent, protect against, control and provide a public health response to the international spread of disease in ways that are commensurate with and restricted to public health risks, and which avoid unnecessary interference with international traffic and trade.” [1]. Article 13 (Public Health Response) of the IHR(2005) obligates States Parties to develop, strengthen and maintain the capacity to respond promptly and effectively to public health risks and emergencies of international concern as soon as possible but no later than five years from the entry into force of the regulations [1]. Progress by States Parties toward the attainment of the stated goals is based on eight core capacities, to be implemented by the year 2012.

Core capacity 8 of IHR(2005) obligates World Health Organization (WHO) Member States to establish mechanisms for providing reliable and timely laboratory identification and characterization of infectious agents and other hazards likely to cause public health emergencies of national and international concern, including shipment of specimens to the appropriate laboratories if necessary. Laboratory services therefore play a major role in all the key processes of the IHR, including detection, assessment, response, notification, and monitoring of events. Developed countries with well-organized routine laboratory services can easily meet this core capacity through existing systems. However, resource-limited countries, especially those in sub-Saharan Africa, need considerable capacity building. Attaining adequate capacities will require functional community, sub-national and national systems. Therefore, laboratory capacity building for IHR core capacity 8 must by necessity focus on strengthening the routine systems within which events are detected and reported.

For a long time, laboratory services were not considered a priority for most resource-limited health care systems in Africa, resulting in poor infrastructure, low human resource capacity and inappropriate technologies [2]. The need for building laboratory diagnostic capacity in Africa has been well articulated over the recent past [2-6]. Consequently, there have been efforts to enhance laboratory capacity over the past five years in many sub-Saharan countries. These efforts have largely been driven by the need for improved laboratory support to surveillance and treatment programs for specific diseases such as HIV/ AIDS, malaria, tuberculosis (TB) and avian influenza [7] at national and a few regional centers. Indeed, a recent review by Olmsted et al (2010) suggests a general improvement in infrastructure and service provision [8]. However, more challenges still exist, including the lack of sufficient numbers of well-trained laboratory scientists in public health service and inadequate laboratory management and leadership skills. There is no or limited distribution of available national laboratory guidelines and a lack of internal and external quality assurance systems, which is usually accompanied by low appreciation of quality control. No efforts have been made towards assisting laboratories to attain international standards such as ISO 15189 and hence slow progress to accreditation. Other challenges have been the poor quality, limited analysis and utilization of laboratory data; inconsistent and inadequate reagent supply chains; and lack of equipment maintenance [9].

The African Field Epidemiology Network (AFENET) is a coalition of Field Epidemiology Training Programs (FETPs) in Africa, initiated in 2005 out of a need to network and facilitate the strengthening of disease surveillance and public health responses to epidemics in the region. Since then, AFENET has grown in membership from four to ten countries. AFENET is organized around a networking and service concept, operating mainly through cooperation with other agencies, host country health ministries, universities, non-governmental organizations (NGOs), the private sector and an extensive network of professionals. Currently, the organization’s activities are focused on six strategic priorities, namely: 1) field epidemiology capacity development; 2) public health laboratory capacity development; 3) public health disease surveillance and effective response; 4) networking and collaboration; 5) AFENET’s institutional development; and 6) information and publications. In this paper, we discuss some of AFENET’s experiences in building laboratory capacity in the region.

## AFENET’s laboratory capacity building eforts

### Human resource development

The capacity to plan and implement an effective public health laboratory system that meets the IHR core capacity requirements depends on the availability of highly-trained and motivated personnel that are not only technically competent, but also possess strong leadership and managerial skills. For years, there has been a lack of advanced-level training programs for laboratory scientists, and this has contributed to the poor state of the laboratory sector in the region. While laboratory staff could be imparted with technical skills, the field has been short of leadership since laboratory staff are rarely included in the decision-making process of health service planning [10].

Since 1993, AFENET founding member countries have been running a graduate-level program in public health, the Field Epidemiology Training Program (FETP), to build a critical mass of public health leaders who are available to respond to public health challenges in the region. Recognizing the effectiveness of this approach in providing competent and motivated staff, a proposal was made to leverage the curriculum by adding a laboratory component (known as the Laboratory Track). This was piloted in Kenya in 2004 as a Field Epidemiology and Laboratory Training Program (FELTP); laying the framework on which similar programs have been founded in other countries.

The generic curriculum for the laboratory track was developed with input from the Kenyan Ministry of Health, universities, and the American Society for Laboratory Medicine, and has since been adapted by other countries. Admission requires a bachelor’s degree in medicine or other related sciences. Although detailed content may differ slightly from program to program, they generally aim at developing a similar set of basic competencies, as detailed by Kariuki et al (2008) [10]. The program combines short didactic courses and laboratory bench work to impart skills in advanced laboratory science; the use of computers in public health; basic and field epidemiology, biostatistics, and public health surveillance; the epidemiology and diagnosis of communicable and non-communicable disease; and management and leadership. This is a two year program with residents spending 65-75% of this time in field placement. Table 1 summaries the current status of residents enrolled or graduated from the Laboratory Track program.

**Table 1:** Residents and graduates of Laboratory Track programs in AFENET member FELTPs

Program	Year Started	Graduates	Current Trainees	Total Enrollment
Kenya	2004	20	12	32
South Africa	2007	0	15	15
Nigeria	2008	0	8	8
Tanzania	2008	0	13	13
Rwanda	2010	0	4	4
WAFELTP*	2010	0	3	3
Totals		20	55	75

*West Africa Field Epidemiology Program, based in Ouagadougou, but serves four French speaking countries – Burkina Faso, Togo, Mali and Niger.

So far, 75 trainees have been enrolled and 20 have graduated from the Laboratory Track in the six FELTPs. To our knowledge, these are the first advanced-level training programs focusing on public health laboratories, combining technical, managerial and leadership skills in this region. Even FETPs without a designated Laboratory Track are contributing by enrolling laboratory personnel for training in Field epidemiology. For example, the Zimbabwe and Uganda FETPs have so far trained six and two laboratory personnel, respectively.

The training is designed to impart a ripple effect throughout the region. This is achieved through three approaches: 1) by utilizing the existing programs to train “seed” personnel from other countries in the region, who will then play a central role in the development of national programs; 2) by encouraging graduates to participate in mentoring programs in other countries; and 3) by having residents and graduates conduct short courses. For example, the Kenya program has trained residents from Ghana, Southern Sudan, Tanzania, and Uganda. The Nigeria, South Africa, and Tanzania pro grams were mentored by graduates of the Kenya program. Ideally, as more countries gain the capacity to run their own FELTPs, there will be an algorithmic growth of trained professionals in the region.

The graduate-level training is complemented by short in-service training courses for mid-level managers and other peripheral laboratory staff to enhance systems for routine laboratory operations in district/provincial levels. The short courses employ two innovative approaches: 1) jointly training medical and laboratory personnel primarily on laboratory matters; and 2) incorporating a 3-month field mini-project with presentations of findings before a panel. Over 500 field staff have been trained in these short courses. The short course curricula are available as open resources, and AFENET provides technical support to any country desiring to conduct these courses at any time [12]. Through the graduate and short course trainings, AFENET is playing a critical role in fast-tracking the building of a sustainable senior-level cadre of laboratory personnel that will champion the attainment and maintenance of IHR core capacities in the region. Moreover, there is evidence that if appropriate steps are taken, the retention rate of these graduates in their countries of origin is quite high [11].

### Laboratory outbreak investigation kit

To enhance timely laboratory support during outbreak investigations, as well as to facilitate the learning of the FELTP trainees, AFENET packages and distributes a comprehensive outbreak response kit to countries where disease outbreaks occur regularly. The contents are adapted according to the specific country’s needs and most frequent diseases. This kit has been provided to Ministries of Health in Uganda, Kenya, Zimbabwe, Ethiopia and Ghana. The generic kit content and guidelines are available at the AFENET website.

### Laboratory quality assurance: The HIV external quality assurance (EQA) project

The unreliability of laboratory results is cited as one of the major limitations to laboratory support for health care in Africa [2]. The utility of laboratory systems in the implementation of the IHR is also greatly dependent on the reliability and quality of laboratory results. However, applicable and cost-effective models for achieving accurate and reliable laboratory results in the low-income African countries have yet to be optimized.

One of the areas where external quality assurance (EQA) is critical is in the monitoring of the routine HIV rapid testing. Most countries in the region depend on re-testing as the primary means of monitoring the quality of HIV serologic testing. This approach is expensive, time-consuming and logistically challenging in decentralized settings [13]. Traditionally, HIV external quality assurance programs use liquid serum or plasma specimens that require special cold storage and transportation conditions. The biohazard risks and costs of this approach have resulted in national quality assurance programs being weak, and consistent quality assurance is generally lacking in lower-tier laboratories [13].

In 2009, AFENET, in collaboration with the U.S. Centers for Disease Control and Prevention (CDC) and the mandated national institutes in Uganda and Tanzania, began a pilot HIV EQA project using the recently developed dried tube specimen (DTS) Proficiency Testing (PT) panels. This approach addressed a number of the technical and logistical challenges of re-testing as reported in the Atlanta and Kenya studies [13]. The DTS is a cost-effective approach and is easy to prepare, stable at high temperatures and can be transported by mail to any part of the country.

Working with CDC, in-country teams and the host country health ministries, 200 laboratory and non-laboratory testing sites in each country were selected. DTS panels are produced by the national reference laboratories and distributed to these sites on a quarterly basis (or depending on country algorithm) through the postal services. After testing at the sites, the results are sent back to the reference laboratories for analysis and scoring. Feedback reports are generated and poorly performing sites are provided with support supervision visits and/or retraining. The project supports the mailing costs, data entry and laboratory scientists to provide technical backup.

Preliminary data received from the first PT round reported 80% and 91% response rates in Uganda and Tanzania, respectively. Reasons for poor performance were identified per sites. Common problem areas include failing to follow national testing algorithms, not mastering proper pipetting technique, failing to follow DTS reconstitution procedures, new or untrained testing staff , and clerical errors in recording test results in both registers and PT report forms. Proper corrective actions, including on-site training and demonstrations, were taken.

This project provides an opportunity to assess parameters for effective EQA systems in Africa, such as local production of EQA materials and the utilization of the local mailing and transport systems. Through this project we hope to identify cost-effective ways of designing and implementing EQA systems, especially for rapid diagnostics that are expected to play an increasing role in the region.

### Basic Laboratory Information Systems (BLIS) project

At the core of IHR practice is reliable information. In most African settings, laboratory data is stored in a format that makes retrieval, analysis and summarization for public health action difficult. Laboratory information is often captured in multiple reports and may be inaccurate, non-standardized and illegible.

The Basic Laboratory Information System (BLIS) is an easy-to-use software that can be used as an effective alternative to paper-based approaches to enhance laboratory data management. As such, labs move a step towards accreditation by using BLIS to improve laboratory information management. Moreover, if properly integrated into the laboratory management practices, BLIS addresses document and record management, inventory control and forecasting. Laboratory sites implementing BLIS receive an initial evaluation prior to installation of the software which is customised to the facility needs. The laboratory receives hardware as well as training. Ongoing information technology support is provided by AFENET personnel. Use and management of laboratory data is assessed by monitoring ease of access and retrieval of patient data, as well as laboratory quality indicators like turnaround time.

Lessons coming from the field indicate that BLIS is making data storage and analysis easier. However, computer literacy at low-tier laboratories remains a challenge. With readily available and timely information, public health responses to diseases of international concern can be guided appropriately.

### Laboratory networking

Where resources are scarce and inequitably distributed, networking may reduce the gaps between different populations. WHO advocates for the establishment of national public health networks to ensure the timely exchange of information and the adequate support of laboratory services at all levels. At both the national and sub-national levels, only a few countries have functional public health laboratory networks in place. In order to strengthen public health capacity within Africa, AFENET created an AFENET-Lab unit to oversee the networking and implementation of laboratory strengthening initiatives. One of the unit’s mandates is to promote public health net working and cross-border collaborations between sub-national, national, regional and inter national public health laboratories and laboratorians for information sharing, technical support, training and other capacity building programs. We believe that this will enhance laboratory capacity by reducing the isolation of peripheral laboratories and enabling them to access network resources. AFENET-Lab began a pilot laboratory net working project in Uganda in 2009, when a memorandum of understanding was signed with the Ministry of Health, Biomedical Laboratory Network (BLN), Uganda Medical Laboratory Technology Association (UMLTA) and Uganda Association of Biomedical Scientists (UABMS).

### Strengthening Laboratory Management towards Accreditation (SLMTA) program

Accreditation is a means of certifying the competence of a laboratory to perform specific types of testing and it enhances customer confidence in accepting testing results. Medical laboratories are accredited by attaining requirements for quality and competency based on international standards, namely ISO 15189. Accredited laboratories therefore play a critical role in providing reliable information to inform IHR decision-making and guiding public health response. Currently, there are very few accredited laboratories in Africa and most of these belong to private sector or international research organizations. The accreditation process requires stringent conditions that have been largely unattainable by most public health laboratories in Africa.

To address the paucity of accredited laboratories in the African region, the WHO Regional Office for Africa (WHO AFRO) established a stepwise approach for laboratories to attain the required standards. This approach supports laboratories at all levels through a series of evaluations using demonstrated improvements, which are recognized and rewarded for the progress [14] [15].

To support this accreditation process, the Strengthening Laboratory Management towards Accreditation (SLMTA) program — a training and mentoring toolkit for laboratory improvement and accreditation was developed. The purpose of the SLMTA trainings is to strengthen laboratory management for immediate and measurable laboratory improvement and to accelerate the process toward lab accreditation by WHO AFRO. AFENET, as a regional partner, took the lead in creating the momentum for the SLMTA roll out update which is posted on the AFENET-Lab website.

AFENET, working with other partners, has so far provided technical assistance by conducting baseline assessments and trainings in Rwanda, Zimbabwe and Swaziland. AFENET is therefore well positioned to support different countries in the roll out of SLMTA.

To garner support for this effort, AFENET collaborated with the WHO, CDC, and the Ugandan Ministry of Health to organize a workshop for senior policymakers to advocate for laboratory accreditation. Over 40 public health experts, including officials from the health ministries of Uganda, Kenya, Tanzania, Rwanda, and Ethiopia met in July 2010 in Kampala. Countries expressed their commitment to develop national laboratory policies and strategic plans that incorporate laboratory accreditation within the next coming year.

### Equipment calibration

Laboratory equipment maintenance and calibration is one of the key components of improving the quality of laboratory services, and is also critical in moving laboratories towards the stepwise WHO AFRO accreditation scheme. Additionally, quality equipment is essential to produce the valid and reliable laboratory results needed to inform decision-making in outbreak situations. The lack of working equipment has affected the effective delivery of health care in resource-poor settings. Often, laboratory equipment does not function properly because regular maintenance services are unavailable, particularly for ancillary equipment. Very little effort has been directed towards laboratory equipment calibration. The few automated laboratory machines that come with service contracts are typically the only equipment that receive regular maintenance. Additionally, the existing commercial services providers have remained costly, making it difficult for health ministries to be able to maintain all the laboratory equipment.

AFENET, in collaboration with CDC and other partners such as Global Health Systems Solutions (GHSS), has organized training sessions to create a pool of local and regional biomedical engineers and equipment technicians able to meet the demands of laboratory equipment maintenance and calibration on a regular basis. This training creates regional capacity that reduces the costs of having the laboratory equipment serviced and calibrated. The trained cadres are part of the health ministry institutions, thereby ensuring that capacity to address equipment calibration is readily available. The biomedical engineers and equipment technicians are trained on servicing and calibration of a variety of laboratory equipment. These cadres are then sent back to their countries to provide equipment servicing and calibration support, particularly to the laboratories enrolled for accreditation.

Six participants, two from each country, from Kenya, Tanzania and Cameroon went through the rigorous practical training in calibration and maintenance of pipettes, microscopes, heating blocks, incubators, autoclaves, microplate washers and readers, vortex stirrer, centrifuges, refrigerators and freezers. Pre- and post-assessment tests were conducted, and results showed an increase in the knowledge gained during the training. Table 2 shows the scores. There is a great need to support these seed staff so they can scale up this training and acquire standardized calibration tools for these countries.

**Table 2:** Pre and post test performance scores of the equipment calibration training

Participant Code	Pre test score (%)	Post test score (%)	Score increase
1.	38	90	52
2.	23	80	57
3.	10	75	65
4.	15	70	55
5.	25	85	60
6.	40	90	50

### Harmonization of laboratory training materials

Until recently, different partners have been conducting various laboratory capacity building trainings in Uganda without following a standard curriculum or using standardized learning materials. A number of development partners have implemented various trainings to build laboratory capacity for health services in Uganda. Consequently, a single laboratory may receive a number of different training materials from various well-meaning organizations, and this can be overwhelming to an already understaffed laboratory. To address this problem, the training sub-committee of the Uganda National Health Laboratory Technical and Advisory Committee (LTC) received financial and technical support from AFENET to put together a comprehensive training package to equip laboratory workers with the management skills they require to be effective in their positions. The training packages are a compilation of learning materials from various partners that were customized to meet the requirements of agreed upon curriculum. The curriculum is practical oriented and targets not only laboratory-specific needs, but also general management skills to achieve overall system strengthening for health services.

## Challenges and opportunities

### Challenges

Decision-making can be an arduous process in AFENET’s large network because of the multiplicity of players and divergent interests. The limited appreciation of the laboratory as a priority area for capacity building slows down the buy-in for various initiatives by national authorities. One of the major challenges AFENET has encountered is limited funding, which often restricts what can be done or is insufficient to maintain an effort till it has reached a sustainable level.

### Opportunities

AFENET and its partners have done a commendable job, and there are still many opportunities for improving the capacity of the laboratory sector. Available funding has so far enabled AFENET to develop and demonstrate effective approaches; however, most of these programs have yet to be implemented. It is AFENET’s vision that every country in the region should have its own FELTP and enhance peripheral personnel skills through short course training. The SLMTA process is also an opportunity to support more laboratories towards accreditation. Laboratory mentoring is also critical especially for improving performance in peripheral laboratories.

In addition, gaps still exist in biosecurity and biosafetly, an area in which AFENET is just beginning to build training capacity. Specifically, there is a need for training on the handling, packaging and shipment of infectious substances. Improving biosecurity is another major opportunity that still exists. Other gaps include programs that build capacity in logistics management, strengthening of national public health laboratory networks, rolling out mid-level manager training and external quality assurance.

## Conclusions

Detection, response, notification and monitoring of PHEICs are the key components of the IHR and all require robust laboratory support. Therefore, capacity building for the IHR must by necessity also focus on strengthening the routine laboratory systems within which events are detected and reported. AFENET is purposefully building capacity that runs through the entire laboratory structure from the lowest level to referral facilities by leveraging on the network approach. We believe this approach will build laboratory capacity at lower levels where these events usually occur, and thus ensure timely and adequate laboratory support. With adequate support, AFENET’s approach provides a logical, sustainable and strategic model with a multiplicative effect that can enable countries in resource limited settings attain and sustain the IHR laboratory capacity (core capacity 8).

## Abbreviations

AFENET: African Field Epidemiology Network; AIDS: Acquired Immunodeficiency Syndrome; BLIS: Basic Laboratory Information Systems; BLN: Uganda Medical Laboratory Technology Association; CDC: Centers for Disease Control and Prevention; DTS: Dried tube specimen; EQA: External quality assurance; FELTP: Field Epidemiology and Laboratory Training Program; FETPs: Field Epidemiology Training Program; GHSS: Global Health Systems Solution; HIV: Human Immunodeficiency Virus; IHR: International Health Regulations; ISO: International Organization for Standards; IT: Information technology; LTC: Health Laboratory Technical and Advisory Committee; NGO: Non-government organization; PT: Proficiency Testing; SARS: Severe Acute respiratory syndrome; SLMTA: Strengthening Laboratory Management towards Accreditation; TB: Tuberculosis; UABMS: Uganda Association of Biomedical Scientists; UMLTA: Uganda Association of Biomedical Scientists; WAFELTP: West Africa Field Epidemiology Program; WHO: World Health Organization; WHO AFRO: World Health Organization Regional Office for Africa.

## Authors’ contributions

MMM contributed towards conceptualization, data collection, drafting and final revisions of the version to be published; NN contributed to the technical content of the information presented, conception and design of the projects, drafting and reviewing the article; DM contributed towards the conceptualization, drafting and revising the article it for important intellectual content; SG contributed towards the intellectual content and final revisions of the version to be published.

## Competing interests

The authors declare that they have no competing interests.
